# Cost-effectiveness protocol for treating adult HIV-infected patients with Kaposi sarcoma in resource-limited settings: a phase III, randomized, open-label, non-inferiority study of paclitaxel and pegylated liposomal doxorubicin

**DOI:** 10.1186/s12962-025-00677-x

**Published:** 2025-11-24

**Authors:** John C. Chapola, Selena L. Kleber, Susan E. Krown, Matthew Painschab

**Affiliations:** 1 UNC Project-Malawi, Lilongwe, Malawi; 2 Department of Health Policy & Management, University of North Carolina, Gillings’ School of Global Public Health, Chapel Hill, USA; 3 Memorial Sloan-Kettering Cancer Center (Emerita), New York, NY USA; 4 Indiana University School of Medicine, Indianapolis, USA

**Keywords:** Kaposi sarcoma, Consortium for Advancing the Prevention and Management of Cancer in People with HIV, Sub-Saharan Africa, Chemotherapy agents, Paclitaxel, Pegylated liposomal doxorubicin, Human immunodeficiency virus, Decision tree model

## Abstract

**Background:**

This paper presents the rationale and plan for a cost-effectiveness analysis conducted alongside an open-label, prospective, randomized, two-arm, multicenter, non-inferiority study by the Consortium for Advancing the Prevention and Management of Cancer in People with HIV (AMC) in sub-Saharan Africa. The study compares two commonly used chemotherapy agents, paclitaxel (PTX) and pegylated liposomal doxorubicin (PLD), administered intravenously with concomitant antiretroviral therapy (ART) for the treatment of adult persons living with Human Immunodeficiency Virus (HIV) (PLWH) with severe Kaposi sarcoma (KS) according to WHO guidelines. The two regimens are commonly used in high-resource settings but have not been formally compared in lower-resource settings.

**Methods:**

This study uses a decision-tree model to evaluate the cost-effectiveness of PTX versus PLD for treating severe KS in adults living with HIV. A health system perspective and a two-year time horizon will be applied. Costs, including medications, labs, and hospitalizations, will be estimated using micro-costing and time-and-motion analyses. Health outcomes will be measured in Quality Adjusted Life years using PROMIS 29 + 2 utility scores. Sensitivity analyses will include Daily adjusted life years and Years of Life Lost.

**Discussion:**

This research will provide valuable insights into the cost-effectiveness of these treatments in managing KS. The results of this analysis will have important implications for healthcare providers and policymakers, offering guidance on the optimal treatment approach for HIV-infected individuals with KS.

**Trial registration:**

This study (NCT05411237) is registered on ClinicalTrials.gov, sponsored by the Consortium for Advancing the Prevention and Management of Cancer in People with HIV. It was first posted on June 9, 2022, with the latest update on January 29, 2025. The trial was prospectively registered before participant enrollment. Estimated primary completion is December 2027, with full completion in September 2028.

## Background

Kaposi sarcoma (KS) is a multifocal, systemic tumor originating from endothelial cells [1]. It presents as four distinct clinical variants: classic KS, endemic African KS, iatrogenic KS associated with immunosuppressive therapy, and KS associated with acquired immune deficiency syndrome (AIDS), commonly known as epidemic KS [1, 2].

Developing effective treatment strategies for KS in adult PLWH is important for several reasons. Firstly, KS ranks among the most common malignancies associated with HIV infection and is driven primarily by the interaction of the immune system and Kaposi Sarcoma-associated herpesvirus (KSHV), warranting unique interventions [3]. It is prevalent in sub-Saharan Africa (SSA) because there is a high rate of co-infection with HIV and KSHV [4]. The compromised immune system of HIV-infected individuals makes them particularly susceptible to KS, which can profoundly affect their quality of life and overall health outcomes [5]. KS can involve multiple organs and tissues, leading to functional impairment and potentially life-threatening complications [6]. By implementing effective treatment strategies, symptoms can be alleviated, disease progression can be slowed, and overall survival rates in this vulnerable patient population can be improved. Additionally, KS-related morbidity and mortality exert a substantial economic burden on healthcare systems, underscoring the need for cost-effective treatment approaches that optimize resource allocation [7]. Hence, the development and implementation of evidence-based treatment strategies for KS in adult PLWH are vital for enhancing patient outcomes, reducing healthcare costs, and improving the overall management of HIV-related complications.

The management of KS typically depends on the clinical presentation, extent of disease, and overall health status of the patient [8]. For localized or limited-stage KS, local therapies such as radiation therapy, intralesional chemotherapy, or cryotherapy may be employed [9]. However, for more extensive or advanced cases, systemic therapy is often necessary [6]. Both PTX and PLD have proven effective in reducing KS tumor burden and improving symptom control [10]. A study conducted in the U.S. by the AMC showed the two treatments had similar efficacy, but the rates of neuropathy and neutropenia were higher in patients treated with PTX [8], leading many providers to prefer PLD for first-line treatment. However, the dosing of PTX in that study was every two weeks. A more recent study conducted by the AMC and the AIDS Clinical Trials Group (ACTG) in SSA and Brazil demonstrated that PTX was well tolerated when administered every three weeks instead of every two weeks and was superior to intravenous bleomycin and vincristine and to oral etoposide [3]. However, a direct comparison with PLD using every three weekly PTX dosing has not been done. In addition, the efficacy and safety of PLD for HIV-associated KS in resource-limited settings has not been systematically studied. Thus, the current protocol, AMC-114, was developed to address this question. Historically, PLD has been considerably more expensive than PTX, although there has been a downward trend in cost with the introduction of generic formulations [11].

The use of PTX and PLD in the treatment of KS in PLWH, particularly in resource-constrained settings, is not without challenges. Both medications can induce significant side effects, such as myelosuppression, neuropathy, and cardiotoxicity [12, 13]. The potential for drug-drug interactions and the need for careful monitoring add complexity to their use [12]. Treatment responses can vary, with some patients experiencing limited or transient benefits [14]. Additionally, the cost of administration of these medications can vary due to differing administration needs (for example, specialized administration sets for PTX), required pre-medications to prevent adverse events, administration time (i.e., labor costs), and treatment of adverse events [15]. Given these factors, it is crucial to measure and compare the outcomes and costs associated with treating PLWH with KS using PTX and PLD. Such analyses can help evaluate the effectiveness, safety, and cost-effectiveness of these treatment strategies, enabling informed decision-making and resource allocation. By quantifying the outcomes and costs, healthcare providers can optimize treatment approaches, considering both clinical efficacy and financial implications, to provide the best care for HIV-infected individuals with KS.

## Methods and analysis

### Study design

We will utilize a two-strategy decision-tree model to measure the outcomes and costs of treating adult PLWH with severe KS receiving ART with either (a) PTX or (b) PLD. Whenever possible, the model and results reported adhere to recommendations of the Second Panel on Cost-Effectiveness in Health and Medicine [16]. We will use a two-year time horizon given the lack of high-quality long-term data on outcomes of HIV-associated KS in this population.

### Target population & subgroups

The target population for this study includes individuals aged 18 years or older who are HIV-positive with histologically confirmed KS. Eligible participants will have either T1 (advanced-stage) KS or T0 (limited-stage) KS causing an adverse effect on quality of life and unresponsive to at least 12 weeks of antiretroviral therapy (ART) [17]. Additionally, participants must meet specific laboratory criteria, including adequate blood counts, liver function, and a Karnofsky performance status of at least 60. Patients with prior chemotherapy or systemic cytotoxic therapy for KS will be excluded. Eligible participants must be on an ART regimen likely to suppress HIV and have measurable KS lesions. Individuals with serious infections, heart conditions, prior immunotherapy, psychiatric illnesses, or compliance challenges will be excluded.

### Setting and location

The study will occur at five sub-Saharan African AMC study sites located in Kenya, Malawi, South Africa, Uganda and Zimbabwe. The sites were selected by the AMC because of their access to the relevant patient populations and their capacity to rigorously conduct cancer clinical trials in PLWH.

### Patient and public involvement

The AMC solicits input from its Global Community Advisory Board (CAB) members, which includes representatives of all the clinical trial sites participating in this study, at all stages of protocol development. CAB members work collaboratively with the AMC’s Patient Outcomes, Survivorship, and Engagement Committee to provide input on informed consent documents, help prepare condensed and easy-to-understand one-page summaries written on an 8th -grade reading comprehension level that provide basic information about the study and are used as protocol recruitment tools and contribute to summaries of study results that are distributed to participating sites. The cost-effectiveness analyses discussed in this manuscript are exploratory studies that do not require additional interaction with or effort by study participants, and specific patient and public input on the cost-effectiveness analyses was not solicited.

### Study perspective

The cost-effectiveness evaluation will be conducted from a health systems perspective, providing a comprehensive analysis of the economic implications and outcomes associated with the interventions of PTX and PLD for KS treatment [18]. By adopting this perspective, the study will not only consider the direct costs and benefits to individuals but also evaluate the broader impact on healthcare systems, resource allocation, and long-term sustainability. This approach enables decision-makers to assess the value and efficiency of the interventions in the overall healthcare system, considering factors such as healthcare utilization, costs, health outcomes, and potential budgetary implications [19].

### Time horizon

The study will evaluate the costs and outcomes of the two intervention treatments over a two-year time horizon. By adopting this time frame, the study intends to offer valuable insights into the economic and clinical implications of these interventions. Some previous studies have reported the three-year survival rates of individuals with KS allowing for provider choice of subsequent therapies but the rates of loss to follow-up are extremely high and high-quality, long-term outcome data for modeling purposes are lacking [20].

### Description of the modeling approach

Decision tree methodology will be incorporated to analyze the cost-effectiveness of the interventions under investigation. Decision trees provide a structured framework for capturing the uncertainty and complexity inherent in healthcare decision-making [21]. By using decision trees, we can visually represent different possible pathways and outcomes of the interventions, considering various treatment options, patient characteristics, and potential events or complications [22]. Decision trees allow us to quantify the probabilities of different outcomes, such as treatment success, adverse events, or disease progression, and estimate associated costs and health outcomes [20, 21]. This enables us to assess the cost-effectiveness of the interventions by comparing their expected costs and benefits over time. Decision trees are particularly valuable in economic evaluation models as they facilitate transparent and systematic analysis, aiding decision-makers in understanding the potential trade-offs and informing resource allocation decisions in healthcare [23].

KS patients on either medication experience different transitional states, outcomes, and paths during their treatment journey. The decision tree captures various possibilities for study volunteers considering their response to the medication and potential outcomes.

### Model structure and disease progression

**Fig. 1:**
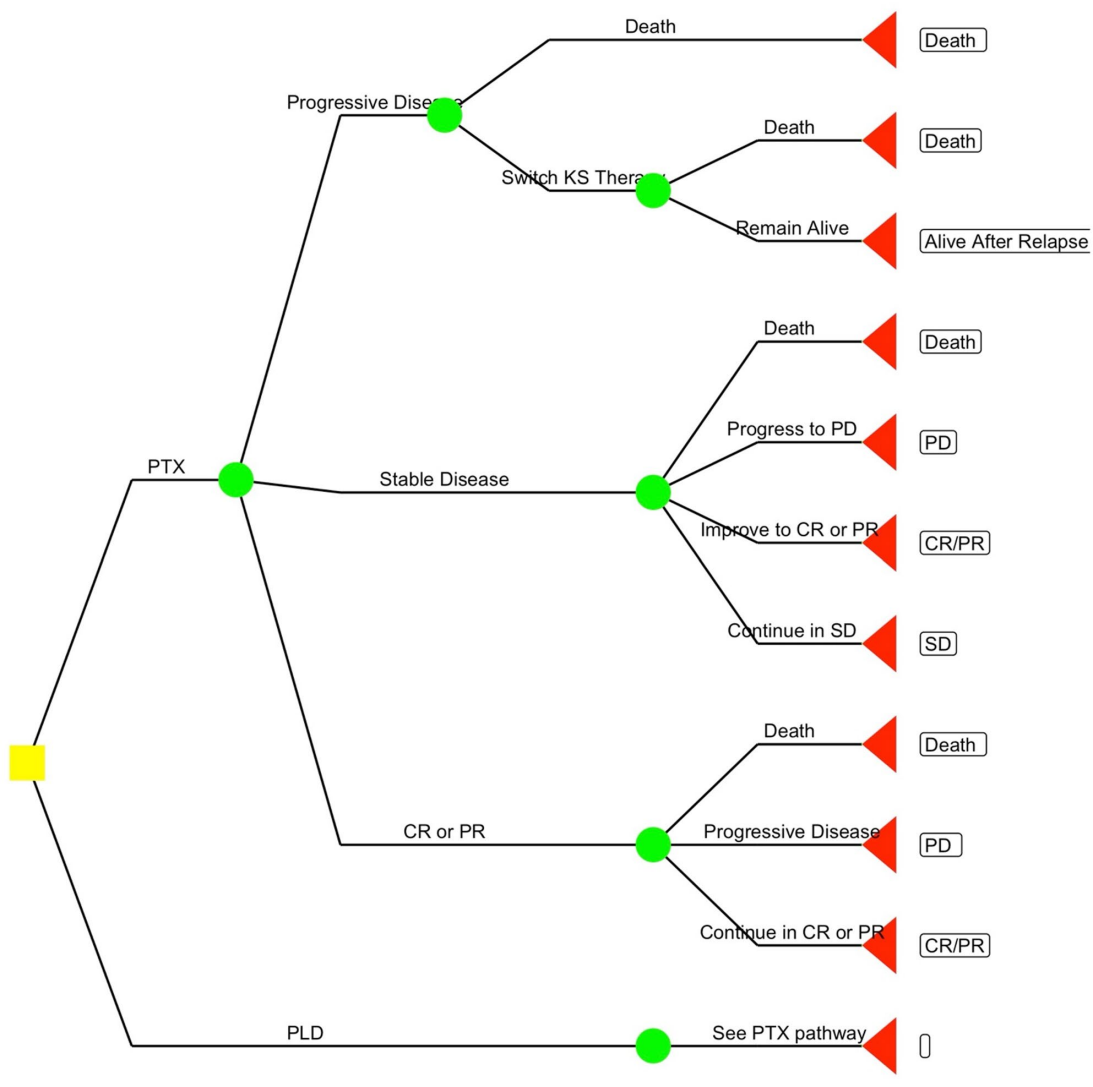
Health economic decision-tree model of participants receiving PTX or PLD. This model represents potential clinical outcomes, resource use, and patient trajectories

Study participants randomized to either treatment arm, PTX or PLD, may achieve different outcomes in terms of disease response. These different outcomes in terms of disease response have been classified using a health economic decision-tree model (Fig. 1), which is a structured framework for modeling possible clinical outcomes, costs, and patient pathways.

Participants can experience a complete response (CR) or a partial response (PR) (Fig. 1). CR is characterized by the absence of detectable disease for at least four weeks, including no tumor-associated edema, with confirmation through biopsies or imaging where necessary. CR must be achieved in both cutaneous and non-cutaneous disease sites with no signs of disease progression. Alternatively, participants may have a PR, where the number or size of lesions decreases by at least 50%, or 50% of raised lesions flatten, with no new lesions or worsening of existing tumor-associated symptoms, lasting for at least four weeks.

Participants who do not meet the criteria for CR or PR but whose disease is neither worsening nor improving substantially will be classified as having stable disease (SD) (Fig. 1).

Progressive disease (PD) is defined as an increase of at least 25% in the size or number of lesions, or the appearance of new lesions or worsening of tumor-associated edema or effusions (Fig. 1). PD may occur in cutaneous or noncutaneous sites, including visceral disease.

Participants with CR, PR, or SD will undergo regular evaluations at four-week intervals until study week 48, and then every twelve weeks for another 48 weeks. If PD occurs at any point, participants will be followed for any additional KS therapy, which may be documented remotely.

This framework captures the complexity of KS patient responses to treatment and provides a structure for monitoring and adapting treatment plans over time. By understanding these potential trajectories, healthcare professionals and researchers can tailor patient care to improve outcomes and personalize therapeutic strategies.

### Micro-costing analysis

Costs will be collected by one primary researcher and coordinated with site-specific staff at each site. Data will be collected directly from actual costs paid for goods and services at each of the participating sites including costs of drugs and supplies purchased and supplied centrally by the AMC. Variable costs will include medications, laboratory tests, transportation reimbursement (when relevant), clinical and laboratory supplies, radiology testing, and hospitalization costs. (Table 1). Costs will be calculated on a per-patient, per-visit basis for the following discrete visit/procedure events: diagnosis, initial assessment, PTX administration, PLD administration, adverse event visit, hospitalization, treatment completion, and follow-up (Table 1). Follow-up will be assumed to occur every three months after treatment completion for a total of two years. Personnel costs will be calculated using time and motion analysis as described below.

To measure the frequency of each type of visit and adverse events, we will use the average number of events over the cohort to calculate a per-patient frequency (e.g., chemotherapy cycles, hospitalizations, and laboratory tests). Additional data from previously published research will be used to supplement the observed data for sensitivity analysis.

### Time and motion analysis

Personnel costs will be calculated using a continuous observation time and motion analysis of personnel involved in each visit type. [7] Time and motion analysis is preferred because the percent effort charged to the study may include research-related tasks that do not directly contribute to the costs of patient treatment in this protocol. This method helps provide a more accurate estimate of the true time dedicated to patient care. Participant enrollment and care are likely to be infrequent compared to all tasks and studies a team member may be working on at each of the study sites. In addition, the percent effort also includes a significant amount of time needed for research tasks such as regulatory tasks, data analysis, manuscript writing, etc. that are not related to patient care.

The time and motion analysis will be conducted by a researcher who will travel to each study site to interview personnel on time spent in activities as well as to observe them in the activities of interest. The number of observations will be determined to achieve sufficient representation and accuracy of the observed behaviors, with a goal of two to three observations per activity at each site [24]. In case of inability to conduct continuous observation time and motion analysis for certain tasks, interviews will be administered to health care providers to understand the time spent on specific tasks. The estimated time spent on specific tasks provided by those interviewed will be used in sensitivity analysis.

The amount of time needed for each task will be multiplied by the local salary for each personnel type involved in the patient care for the study. Salaries and fringe benefits of the organizations conducting the study will be used in each case, although an attempt will also be made to establish the usual local salary in government facilities at each location for sensitivity analysis. A list of personnel to be evaluated at each local clinic site is listed in Table 1.

### Outcome measures

Quality Adjusted Life Years (QALYs) will be calculated using the QALY data from the PROMIS preference scoring system (PROMIS 29 + 2) [25] in each local language, the response status, and the overall survival time seen in each study arm. The PROMIS 29 + 2 system gives a single number that is the health-related quality of life of the participant at the time of the survey with a value of zero equating to dead and one equating to perfect health [26]. QALYs are calculated by multiplying the utility value by the amount of time in that utility level and will be assessed at initial diagnosis, mid-treatment, end-of-treatment, six months after treatment, and relapse. The conversion from the PROMIS 29 + 2 to QALYs for each participant will be derived from the standard values from a large survey of the valuation of health outcomes in the United States [25]. Admittedly, there are cultural differences in preferences QALYs between SSA and the United States; however, to our knowledge, there are few preference-based surveys that have been conducted in healthy populations in SSA upon which to make the same QALY conversions and the incremental effects of these preferences are likely to be marginal. Furthermore, because both study arms involve participants from the same population, variation in cultural or regional health-state preferences is not expected to affect the *differential* QALY estimates between treatment groups. Therefore, the PROMIS preference weights derived from healthy populations are considered appropriate for both arms though this will be a limitation in the analysis.

Years of life lost will be calculated using the age of the participant at the time of death and expected life expectancy in the country of the participant from the WHO life Table [27].

Disability-adjusted life years (DALYs) will also be used in sensitivity analysis. DALYs will be derived from the values used for other cancers as there is no specific disability weight for KS [28]. DALYs are commonly used in cost-effectiveness evaluations in low- and middle-income countries (LMICs) to compare cost-effectiveness across different health interventions as QALYs are frequently not available. DALYs are calculated as the sum of the years of life lost due to premature mortality (YLLs) and the years of healthy life lost due to disability (YLDs). One DALY represents the loss of the equivalent of one year of perfect health.

### Costing and model assumptions

Overhead costs (laboratory and hospital infrastructure) will not be collected or used in the analysis as they will not be different between the two arms studied. The exception is that an estimation of hospital room costs will be included in the hospitalization costs in case of differences in the frequency of hospitalization between the two arms.

All participants in this study will already be receiving ART as part of standard HIV care, in accordance with national and WHO guidelines. Because ART use and associated adherence support are identical across both treatment arms, ART-related costs will not be included in the incremental cost-effectiveness analysis. While ART adherence is an essential component of KS management, it is not expected to differentially influence cost or effectiveness outcomes between the paclitaxel and liposomal doxorubicin arms. Nevertheless, we acknowledge its clinical importance and will discuss potential implications of ART adherence variability in sensitivity analyses where relevant.

Costs and outcomes will be discounted at 3% annually and varied from 0 to 6% for sensitivity analysis. Given the multiple branches and subgroups in the decision-tree model, we will use probabilistic sensitivity analysis with 1000 Monte Carlo simulations to ensure stable estimates of costs and QALYs. Confidence intervals around incremental cost-effectiveness ratios will be assessed to ensure sufficient precision. The sample size of the trial informing model inputs will be considered to support adequate power for detecting clinically meaningful differences, and sensitivity analyses will explore the impact of uncertainty in key parameters.

Probabilities and timing of key events for each branch of the decision-tree model will be specified based on literature, expert opinion, and available trial data. These assumptions will be explicitly documented and incorporated into the probabilistic sensitivity analysis. Event timing will reflect expected durations for treatment response, follow-up, and disease progression, aligned with the current study timeline—with accrual anticipated to begin in the first quarter of 2026, continue for approximately two years (through 2028), and follow-up expected to conclude in 2029. This approach allows the model to handle uncertainty and missing data appropriately.

Costs will be collected in local currency at each site and will be adjusted to USD using the average exchange rate for the year of cost collection. Adjustments for inflation to 2024 USD will be made using the World Bank GDP deflator for each country if costs are collected in a different year. Costs will also be converted to international dollars to provide additional context given the differences in the cost of living between countries. International dollars are calculated with the typical USD amount for tradeable goods but require conversion of the cost of non-tradeable goods (blood transfusion, personnel, hospital room, and transportation) using the purchasing power parity of the local currency for the year of interest (2024).

### Outcomes

The primary outcome of interest for the economic analysis will be the incremental cost-effectiveness ratio (ICER) as measured in cost in US dollars (USD) per QALY gained.

Additional exploratory outcomes of interest include:

- ICER of cost in USD per completion of 2 years without death or relapse (given 2-year event-free survival is the primary AMC-114 study outcome).
- ICER of cost in USD per life years gained.
- ICER of cost in USD per DALY averted.
- ICER of cost per each of the above outcomes with costs calculated in international dollars.

### Policy and equity implications

The findings from this cost-effectiveness analysis will provide evidence to support national decision-making related to cancer treatment procurement, formulary inclusion, and resource allocation in sub-Saharan Africa. By quantifying the incremental cost per QALY gained and DALY averted for paclitaxel and pegylated liposomal doxorubicin, this study will generate data that can guide policymakers in prioritizing treatments within limited health budgets. The results may also inform equitable access strategies for HIV-associated malignancies, particularly Kaposi sarcoma, by identifying cost-effective treatment options that balance affordability and clinical benefit across different health system settings.

## Discussion

This cost-effectiveness protocol is important for several reasons. KS is a common malignancy in individuals with HIV infection, particularly in resource-limited settings where access to effective treatments may be limited. Resource-limited settings refer to regions or areas where there is a scarcity of financial, infrastructural, and human resources necessary to provide optimal healthcare services [29]. Cost-effectiveness and budget impact analyses are critical for policymakers and government officials to prioritize various health interventions in severely resource-constrained systems. In the context of KS treatment, these settings are often characterized by limited access to diagnostic tools, medical facilities, medications, and trained healthcare professionals. Due to financial constraints, healthcare systems in resource-limited settings may struggle to afford or procure expensive chemotherapy drugs or innovative treatment modalities [30]. Additionally, the lack of proper infrastructure and equipment, such as laboratories and imaging facilities, can hinder accurate diagnosis and monitoring of KS patients [28, 29]. Moreover, a shortage of healthcare workers and their limited expertise in managing KS can impede timely and appropriate treatment [31]. These challenges collectively contribute to the limited availability and accessibility of effective KS treatments, making individuals affected by KS in resource-limited settings particularly vulnerable to suboptimal care and poor health outcomes.

AMC-114 aims to evaluate two chemotherapy drugs, PTX and PLD, in treating KS in such settings. The assessment of cost-effectiveness for PTX and PLD in the study is important as it can help determine the most feasible and efficient treatment option in resource-limited settings. By evaluating the costs associated with each treatment, including the expenses of medications, administration, monitoring, and management of side effects, the study can shed light on which option offers the best value. Furthermore, it can help identify any disparities in access and affordability between the two treatments, ensuring that decision-makers in resource-limited settings can allocate funds effectively. A retrospective analysis of previously published clinical trials data by Freeman et al. modeled the cost-effectiveness of four chemotherapy regimens for advanced AIDS-associated Kaposi sarcoma in Kenya and concluded that PTX was the most cost-effective approach. The authors suggested that the results could be generalizable to a range of LMICs across SSA [32]. However, the limitations of that study included reliance on risk ratios from U.S.-based trials due to the lack of direct comparison data in LMICs, and the absence of measures of quality-of-life, micro-costing or time and motion, and operational costs of implementing new chemotherapy regimens, highlighting the need for more comprehensive data collection in future research [32]. A prospective study, like our planned open-label, randomized trial in SSA, could address these gaps by providing more robust and context-specific conclusions on cost-effectiveness and quality-of-life outcomes.

There are several limitations to consider when assessing the cost-effectiveness of PTX and PLD. Firstly, the cost-effectiveness analysis heavily relies on the availability of accurate cost data, which can be challenging to obtain in resource-limited settings [33]. Limited access to comprehensive cost information, including drug prices, administration costs, and supportive care expenses, may affect the accuracy and generalizability of the results. Additionally, the study’s time horizon may not capture long-term treatment outcomes or potential late effects, which could lead to an incomplete understanding of the true cost-effectiveness of these treatments.

Regarding the use of time and motion analysis, there are also limitations to consider. Time and motion analysis involves observing and analyzing the time required to complete specific healthcare tasks [34]. One limitation is that it may not capture the full complexity of clinical workflows, as some tasks or activities may not be easily measured or observed. The analysis may also be subject to observer bias or errors in data collection, impacting the accuracy and reliability of the results. Furthermore, time and motion analysis typically focuses on specific healthcare processes, potentially overlooking broader systemic factors that influence resource utilization and efficiency. Lastly, the findings of time and motion analysis conducted in one setting may not be directly applicable to other healthcare contexts, due to variations in resource availability, healthcare practices, and patient populations. Overall, while both cost-effectiveness analysis and time and motion analysis provide valuable insights, it is important to acknowledge their limitations and interpret the findings cautiously, considering the specific context and potential biases associated with each approach.

## Appendix

**Table 1:** Table of each of the data types to be collected and the comprehensive list of each. Within each generic cost category, several specific data items will be collected such as “paclitaxel” and “doxorubicin” within chemotherapy

Data type	List of items in data type
Visit type	Diagnosis
	Enrollment
	Paclitaxel administration
	Liposomal doxorubicin administration
	Adverse event visit
	Hospitalization
	Completion
	Follow up
Generic cost categories	Chemotherapy
	Supportive care drugs
	Laboratory
	Supplies
	Blood bank
	Pathology
	Radiology
	Personnel
	Hospital room
	Transportation reimbursement
Personnel	Provider (Medical officer or clinical officer or oncology specialist)
	Nurse
	Pharmacist
	Pathologist
	Laboratory technician

## Data Availability

The data supporting the findings of this study are not publicly available due to considerations related to participant privacy and confidentiality, but can be made available upon reasonable request from the corresponding author. Requests will be evaluated according to ethical guidelines and institutional policies to ensure the responsible sharing of research data while safeguarding the anonymity and well-being of study participants participants.

## Abbreviations

ACTG: AIDS Clinical Trials Group
AIDS: Acquired immune deficiency syndrome
AMC: Consortium for Advancing the Prevention and Management of Cancer in People with HIV
ART: Antiretroviral therapy
CAB: Community Advisory Board
CR: Complete response
HIV: Human Immunodeficiency Virus
KS: Kaposi Sarcoma
KSHV: Kaposi Sarcoma-associated herpesvirus
PD: Progressive disease
PLD: Pegylated liposomal doxorubicin
PLWH: Persons living with Human Immunodeficiency Virus
PR: Partial response
PTX: Paclitaxel
SD: Stable disease
